# Mucosal Microbiome in Patients with Early Bowel Polyps: Inferences from Short-Read and Long-Read 16S rRNA Sequencing

**DOI:** 10.3390/cancers15205045

**Published:** 2023-10-19

**Authors:** Zoe Welham, Jun Li, Alexander F. Engel, Mark P. Molloy

**Affiliations:** 1 Bowel Cancer and Biomarker Laboratory, School of Medical Sciences, The University of Sydney, Sydney 2065, Australia; zwel5671@uni.sydney.edu.au (Z.W.); jun.li2@sydney.edu.au (J.L.); 2 Colorectal Surgical Unit, Royal North Shore Hospital, Sydney 2065, Australia; alexander.engel@sydney.edu.au; 3 Sydney Medical School, Faculty of Medicine and Health, The University of Sydney, Sydney 2050, Australia

**Keywords:** bowel polyps, gut microbiome, 16S rRNA sequencing, PacBio long-read sequencing

## Abstract

**Simple Summary:**

Changes in the gut microbiome are associated with bowel cancers, however, less is known about the microbiome during the pre-cancerous bowel polyp stage. This study compared two DNA sequencing technologies to identify the gut microbiome from mucosa of colonoscopy patients diagnosed with bowel polyps or without bowel polyps. We found that different sequencing technologies and bioinformatic pipelines impact on bacterial taxonomy assignments. Overall, there were only minor differences in gut microbiome communities between participants with bowel polyps and those without. However, *Ruminococcus gnavus*, a bacteria commonly associated with inflammatory bowel disease (IBD) was shown to be more abundant in participants with polyps, despite participants with IBD being exclude from this study. This paper adds to our knowledge of the gut microbiome associated with bowel neoplasia.

**Abstract:**

Numerous studies have correlated dysbiosis in stool microbiota with colorectal cancer (CRC); however, fewer studies have investigated the mucosal microbiome in pre-cancerous bowel polyps. The short-read sequencing of variable regions in the 16S rRNA gene has commonly been used to infer bacterial taxonomy, and this has led, in part, to inconsistent findings between studies. Here, we examined mucosal microbiota from patients who presented with one or more polyps, compared to patients with no polyps, at the time of colonoscopy. We evaluated the results obtained using both short-read and PacBio long-read 16S rRNA sequencing. Neither sequencing technology identified significant differences in microbial diversity measures between patients with or without bowel polyps. Differential abundance measures showed that amplicon sequence variants (ASVs) associated with *Ruminococcus gnavus* and *Escherichia coli* were elevated in mucosa from polyp patients, while ASVs associated with *Parabacteroides merdae*, *Veillonella nakazawae*, and *Sutterella wadsworthensis* were relatively decreased. Only *R. gnavus* was consistently identified using both sequencing technologies as being altered between patients with polyps compared to patients without polyps, suggesting differences in technologies and bioinformatics processing impact study findings. Several of the differentially abundant bacteria identified using either sequencing technology are associated with inflammatory bowel diseases despite these patients being excluded from the current study, which suggests that early bowel neoplasia may be associated with a local inflammatory niche.

## 1. Introduction

DNA sequencing of the bacterial 16S rRNA gene supports a growing body of literature reporting gut microbiome differences between colorectal cancer (CRC) patients and healthy controls (for a review, see Rebersek [1]). In limited cases such as *pks+ E. coli* [2] or *Fusobacterium nucleatum* [3], there is a mechanistic causal relationship between these microbes and CRC; however, the roles of specific bacteria or microbial communities contributing to early bowel neoplasia are less clear. Most studies to date have examined the gut microbiome by employing 16S rRNA amplicon sequencing of fecal samples as a convenient, accessible proxy for the gut environment. While stool samples are non-invasive and have diagnostic potential, the microbial composition of the bowel mucosa differs significantly from microbiota detected in stool [4,5,6,7]. Arguably, more informative biological insight can be gained from an ex vivo analysis of gut mucosal biopsies.

The microbiome analysis of mucosal biopsies from individuals with bowel polyps compared to polyp-free controls has highlighted the importance of some microbes. Proteobacteria, in particular the genera *Escherichia* and the closely related *Shigella*, were found enriched in biopsies from adenoma cases compared to free controls [8,9]. *Pseudomonas* [10,11,12], *Enterobacteriaceae* and *Enterococcus* [10,12] and *Helicobacter* [11] were also reported to be more abundant in adenoma cases. Advanced adenomas showed an enrichment of bacteria from the families *Halomonas* and *Oceanospirillales* [13]. Bacteria from the order *Lactobacillales* have also been reported to be enriched in adenoma cases, including *Lactococcus* [10,11] and *Lactobacillus* [11]. *Fusobacterium* sp., which are relevant in advanced bowel cancers, have also been identified as present in polyp cases [14,15]. Conversely, bacteria from the Firmicutes phylum tend to be depleted in adenoma mucosal samples compared to controls, including *Faecalibacterium*, *Blautia* [10,13,16] and *Coprococcus* [5,13]. Bacteria from the Actinobacteria phylum—for example, *Bifidobacterium* [16]—have also been shown to be depleted in adenoma cases.

The variability in findings across cohorts remains a central problem in microbiome analysis. Contributing technical factors include which 16S rRNA variable region is chosen for sequencing and taxonomy assignment, differences in bioinformatic pipelines, and various reporting thresholds [17]. While the short-read sequencing of 16S variable regions can identify ASVs to the family and genus taxonomy level, far fewer ASVs can be assigned at the species level. This limitation contributes to study inconsistencies, as different species or strains from the same genera can have different effects on their environment. For example, *Escherichia coli*, normally harmless and even a beneficial vitamin K producer, also comprises enterotoxic and uropathogenic strains, and these cannot be differentiated from short-read sequencing [18]. Long-read 16S rRNA sequencing is an emerging technique currently supported by Pacific Bioscience (Pacbio) and Oxford Nanopore Technologies (ONT). These methods can sequence large stretches of DNA that encompass the entire 16S rRNA gene (~1500 bases), thus providing higher resolution to assign microbial identity to the species or often strain level.

As an example relevant to bowel neoplasia, Wei and colleagues [19] used both 16S rRNA short-read (MiSeq, Illumina) and full-length 16S rRNA long-read platforms (MinION, ONT) to compare the stool microbiotas of 43 individuals with adenomatous polyps and 53 healthy subjects along with 36 individuals with a positive immunochemical fecal occult blood test (iFOBT). Their LEfSe differential abundance analyses for short- and long-read technologies were highly similar. They found a significantly increased relative abundance of *Fusobacterium mortiferum*, *Fusobacterium varium*, and *Klebsiella pneumonia* in patients with adenomas compared to healthy subjects, while *Blautia luti*, *Bacteroides plebeius*, and *Prevotella copri* were enriched in the gut microbiota of the healthy group. 

Taylor and colleagues [20] compared short-read V3–V4 region sequencing and MinION long-read sequencing of the 16S rRNA gene from biopsies taken directly at the gut mucosa from 11 CRC patients. Unlike Wei and colleagues, they found only 67.6% concordance between bacteria identified at the phyla level and 19.5% at the species level. Both sequencing platforms associated *Bacteroides fragilis*, *Fusobacterium nucleatum* and *Prevotella intermedia* with CRC patients, whereas 81 species implicated in CRC such as *Bacillus*, *Burkholderia*, *Streptomyces* and *Pseudomonas* were found in MinION sequencing alone. Nine species such as *Sphingobium* were unique to 16S short-read rRNA amplicon sequencing. 

These studies examined stool samples from adenomatous polyp patients and tumor biopsies of CRC subjects. Less is known about microbial communities surrounding bowel polyps, and adding to this literature is essential, as bowel polyps are the benign precursor lesions of CRC. This study deployed both short-read (SR) (V3–V4) and Pacbio long-read (PB-LR) (V1–V9) sequencing of the 16S rRNA gene to examine the microbial composition of the gut mucosa in colonoscopy patients with early bowel polyps compared with polyp-free patients. 

## 2. Materials and Methods 

### 2.1. Ethics Statement

This project collected biopsy specimens from participants undergoing scheduled colonoscopies at the Royal North Shore Hospital, Sydney, Australia from 2020 to 2023. Human research ethics (2019/ETH00301) and governance (2019/STE10535) approval was obtained from the Northern Sydney Local Health District. Written informed consent was obtained from all participants. All study samples and information collected were de-identified by assigning a unique study identity code.

### 2.2. Inclusion and Exclusion Criteria

Participant inclusion criteria included the following:

1. Adults *≥* 18 years undergoing colonoscopy for either a positive fecal occult blood test, positive family history of sporadic bowel neoplasia, rectal bleeding, or a scheduled colonic surveillance.
2. Written consent to collect tissue for research purposes and to complete a questionnaire on family medical history and general lifestyle choices.
3. Adequate tissue for research collection and pathological analysis as specified by the treating pathologist.

Exclusion criteria included the following:

1. Patients diagnosed with hereditary bowel polyposis diseases including Lynch syndrome (HNPCC) and familiar adenomatous polyposis (FAP).
2. Patients with Crohn’s disease, ulcerative colitis, active diverticulitis, or other inflammatory bowel diseases.
3. Any condition compromising the patient’s ability to give informed consent or patients who did not consent to the return of incidental findings from molecular analyses.
4. Patients who had taken antibiotics up to four weeks before the colonoscopy.

### 2.3. Specimen Collection

Colorectal polyps were removed as standard clinical care during the colonoscopy procedure. For each polyp removed, two further 2 mm^3^ mucosal biopsies 20 mm and 50 mm adjacent to the polyp were collected. If no colon polyps were detected, two biopsies taken from the proximal and distal colons were collected to form a polyp-free control cohort. All samples were immersed in dry ice at the clinic and then stored at −80 °C. Each polyp sample underwent histopathology assessment by a certified pathologist with findings reported synoptically according to the Royal College of Pathologists of Australasia-authored *Polypectomy and Local Resections of the Colorectum Structured Reporting Protocol* 2nd Edition. 

### 2.4. Bacterial DNA Extraction

DNA was extracted after bead-beating mucosal biopsies adjacent to polyps located in the proximal bowel using the Invitrogen PureLink Microbiome DNA Purification kit (Thermofisher Scientific, Waltham, MA, USA) according to the manufacturer’s instructions. In summary, bacterial cells were lysed in buffer; then, samples were heated at 65 °C for 10 min and underwent bead beating at 50 Hz for 10 min. After centrifugation, the DNA from the samples was bound to a kit column and washed with an ethanol solution. The bound DNA was eluted with Tris buffer pH 8.0. DNA quantity and purity was assessed using a Nanodrop spectrophotometer. All extracted DNA was stored at −80 °C.

### 2.5. 16S rRNA Gene Sequencing and Bioinformatic Analyses

#### 2.5.1. Short Read Sequencing

Fifty-four samples underwent 16S rRNA gene sequencing at the Australian Genome Research Facility (AGRF) in Melbourne. The primer pair 341F CCTAYGGGRBGCASCAG and 806R GGACTACNNGGGTATCTAAT was used to target the V3–V4 region of the 16S rRNA gene. Paired-end sequencing (2 × 300 bp) was performed using the Illumina Miseq platform (Illumina, San Diego, CA, USA). The resulting data underwent a quality check using FastQC [21] and bioinformatics processing using QIIME2 [22]. 

DADA2 [23] was used to trim the reads of the adapter and primer sequences, which were truncated to lengths 270 (forward reads) and 205 (reverse reads) to minimize the effect of low-quality reads. The paired end reads were merged and then filtered for phiX and chimeric sequences. Reads that shared >97% identity with human sequences were removed. All short reads were classified to the lowest possible taxonomic rank by using the QIIME2 q2-feature-classifier plugin to train the Naive Bayes classifier, using the Silva 138 99% OTUs full-length sequences database [24] and the primer pair 341F and 806R, which were used to sequence the V3–V4 16S rRNA region. An amplicon sequence variant (ASV) table was constructed showing the read counts for each identified ASV. 

#### 2.5.2. PacBio Long-Read Sequencing

The fifty-four biopsies underwent long-read 16S rRNA gene sequencing at the AGRF. The primers AGRRTTYGATYHTDGYTYAG and YCNTTCCYTYDYRGTACT were used to sequence the 16S-ITS-23S region, and the resulting sequencing amplicons underwent bioinformatics processing using the Shoreline Biome bioinformatics workflow, which utilizes the proprietary *SB Analyzer* software Version 3.0 as well as DADA2 and QIIME2. In addition, the primers 27F:AGRGTTYGATYMTGGCTCAG and 1492R: AAGTCGTAACAAGGTARCY were used to target the V1 to V9 region of the 16S rRNA gene, and the resulting sequencing amplicons underwent bioinformatics processing using the PacBio workflow at https://github.com/PacificBiosciences/pb-16S-nf (accessed on 25 August 2022). To test the specificity of the two primer pairs, five random samples from the cohort along with control DNA with low human DNA content provided by PacBio then underwent PCR amplification at PacBio to identify any off-target DNA amplification. 

### 2.6. Taxonomy Assignment

To ensure the short-read and long-read data were as comparable as possible, the short-read data were also classified using the GTDB and RefSeq databases along with the Silva database, using the databases and approach outlined by Pacbio (https://github.com/PacificBiosciences/pb-16S-nf/tree/main/scripts (accessed on 26 July 2023), *dada2_assign_tax.R*). Given that the results for the integrated taxonomy databases were comparable to the Silva-only analyses, with improved taxonomic resolution, this approach was used in further analyses. The SR and PB-LR three-database integrated taxonomy data were manually curated to ensure all genera were consistent in their taxonomy assignments, using the NCBI taxonomy browser to standardize discrepancies.

### 2.7. Statistical Analysis

#### 2.7.1. Sequencing Quality

Descriptions of the short-read and long-read sequencing quality, including the total number of reads in each individual sample, read quality, and depth of coverage obtained by SR and PB-LR sequencing, were obtained from QIIME2 workflows.

#### 2.7.2. Alpha Diversity Analyses

Shannon’s index was calculated on the raw ASV counts tables for both SR and PB-LR data sets. This index incorporates phylogenetic relationships between features to produce a qualitative measure of community richness and evenness within each sample. The Chao1 richness index and Pielou’s evenness index were also included as complimentary measures. Wilcoxon non-parametric rank-sum tests for comparing differences between patients with polyps and those without polyps were included, using the R package *ggpubr* (Version 0.6.0). 

#### 2.7.3. Beta Diversity Analyses

The SR and PB-LR data sets were filtered to retain those ASVs that were present at greater than 10 reads (SR) or 2 reads (PB) in more than 10% of total samples and normalized by transforming the data into relative abundances. Beta diversity was assessed by computing Bray–Curtis dissimilarity distances and visualized with a principal coordinate analysis (PCoA) plot.

### 2.8. PERMANOVA

Permutational Analysis of Variance (PERMANOVA) using the *adonis2* function was performed on both data sets to assess whether polyp status (polyp-free versus polyp-associated) significantly contributed to the variance in the data, accounting for age, gender and BMI. The homogeneity of group variance was tested with the *betadisper* function. All diversity measures and PERMANOVA analyses were calculated using the *R* package *vegan* (Version 2.6-4) [25] in the *R* statistical platform.

### 2.9. Differential Abundance Analyses

Both Linear discriminant analysis Effect Size (LEfSe) [26] and Analysis of Compositions of Microbiomes with Bias Correction (ANCOM-BC, Version 2.0.2) [27] were employed to identify differentially abundant ASVs associated with polyp status. LEfSe was employed using the default parameters, while the ANCOMBC model was fit with age, gender and BMI as covariates. Results were considered significant at a *p*-value < 0.05. 

### 2.10. Multivariate Analyses

Due to the interconnected nature of microbiota, further multivariate analyses were performed to assess for bacteria that may be correlated and thus show similar patterns between polyp or polyp-free cases. Principal Components Analysis was employed to identify the main sources of variation in the data, including outliers and batch effects. A supervised sparse Partial Least Squares Discriminant Analysis (sPLS-DA) was used to identify ASVs that were best able to distinguish between polyp-associated and polyp-free cases. All analyses were conducted using the *R* package *mixOmics*, Version 6.22.0 [28].

## 3. Results

### 3.1. Participant Clinical Data

A total of 18 male and 9 female patients with low-grade colorectal polyps and 27 polyp-free control patients matched for age, gender and BMI were included in the study. Twenty-six polyps were adenomatous (tubular or tubulovillous), and one was a sessile serrated lesion. There were no significant differences in age (*p* = 0.11), gender (*p* = 0.9), BMI (*p* = 0.2) or indication for colonoscopy (*p* = 0.7) (Table 1) between the two groups.

### 3.2. Comparison of Long-Read Primer Sets

**Figure 1:**
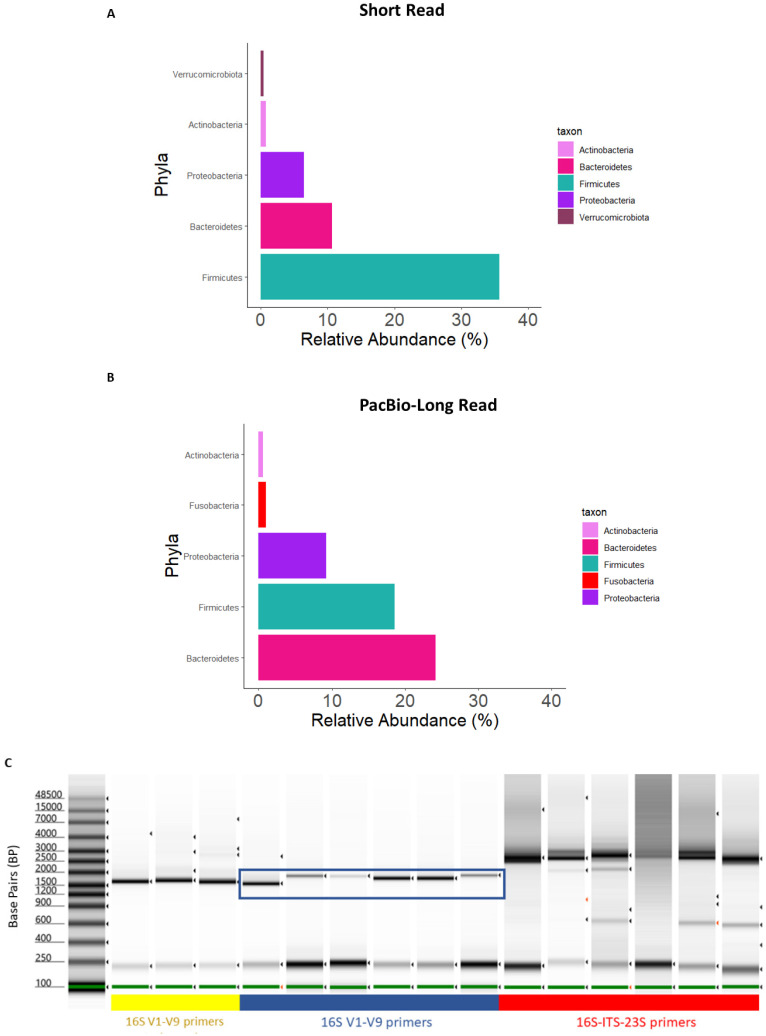
Composition plots aggregated to the phylum level for (**A**) SR and (**B**) PB-LR data. (**C**) Gel electrophoresis of DNA from mucosa samples amplified with bacterial 16S-ITS-23S or 16S V1–V9 primers. Lane 1: DNA ladder. Lanes 2–4 (yellow underline) represent PCR products from stool amplified with 16S V1–V9 primers. Lanes 5–10 (blue underline) represent the PCR products from a selection of mucosal biopsy study samples amplified with 16S V1–V9 primers. Lanes 11–16 (red underline) represent PCR products from a selection of mucosal biopsy study samples amplified with 16S-ITS-23S primers. The 16S rRNA gene amplicon of interest is shown with the blue box.

To test the long-read 16S rRNA gene sequencing method with DNA extracted from mucosal specimens, we initially employed primers to amplify the 16S-ITS-23S (~4300 bp) genomic region, which would enable a high-resolution bacterial taxonomy assignment beyond species-level descriptions. However, a composition plot of the data set at the phylum level of taxonomy showed biases in composition compared with the 16S V3–V4 short-read data and the established literature [29,30] (Figure 1A,B). We made a comparison of PCR products from the 16S-ITS-23S primers against the amplicon obtained using 16S V1–V9 primers (~1500 bp). This revealed that the 16S-ITS-23S primers amplified off-target human DNA that is highly abundant in mucosal samples compared to stool samples (Figure 1C). The 16S V1–V9 primers amplified fewer off-target PCR products and were therefore employed for long-read sequencing of mucosal samples.

### 3.3. Comparison of Short-Read and Long-Read Sequencing Quality

**Figure 2:**
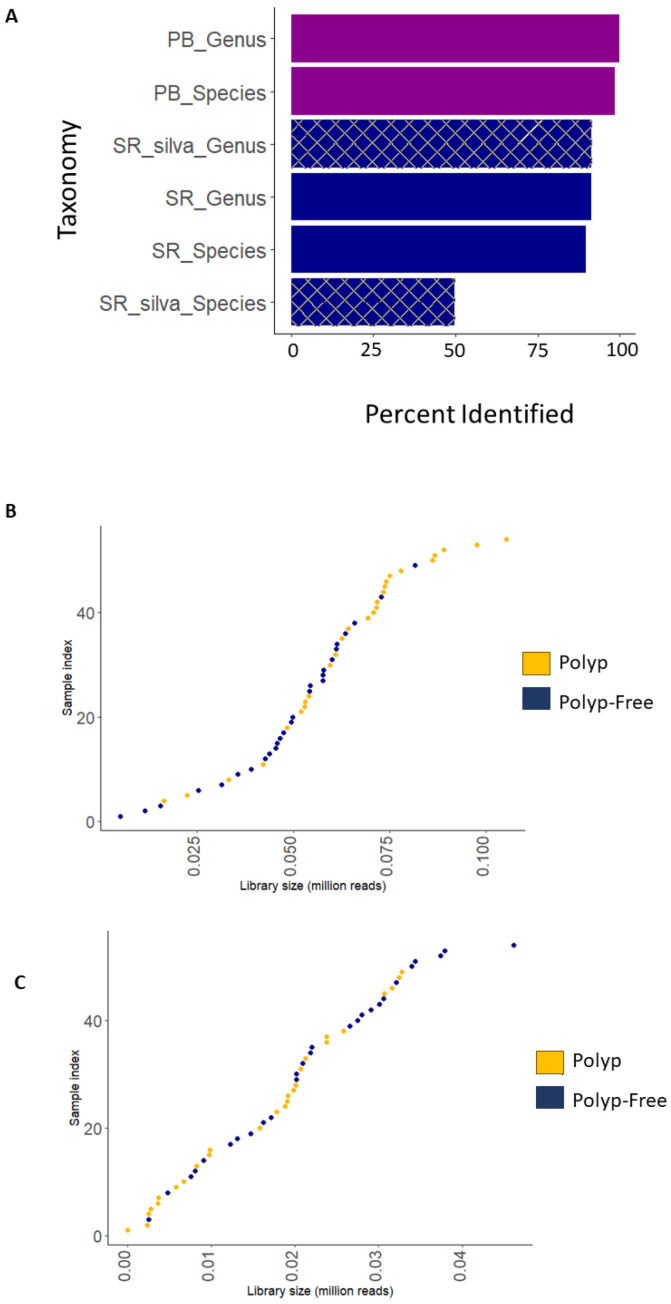
(**A**) Bar chart depicting the percentage of ASVs assigned to genus and species-level taxonomy for short-read (SR), SR silva database only, and PacBio (PB) long-read (LR) platforms. Histograms for (**B**) SR and (**C**) PB-LR library sizes for 27 polyp-associated and 27 polyp-free samples.

This study employed both V3–V4 16S rRNA short-read (MiSeq, Illumina, San Diego, CA, USA) and V1–V9 16S rRNA long-read (Pacbio, Menlo Park, CA, USA) sequencing using the same DNA extracts. Both methods employed independent bioinformatic processing pipelines; however, both used the QIIME2 platform with DADA2 for quality filtering. Sequencing quality results are shown in Table 2. The SR data contained on average 4.25× more reads than the PB-LR data; however, the PB-LR data showed a greater average of reads per sample after DADA2 filtering. The PB-LR data classified 91% ASVs to the genus level and 90% to the species level compared to 88% to the genus level and 50% to the species level using the SR silva database. The number of ASVs assigned taxonomy at the species level from SR data could be improved when using the three taxonomy databases (Figure 2A). Figure 2B,C show histograms of the library sizes for the SR and PB-LR data sets, respectively.

**Figure 3:**
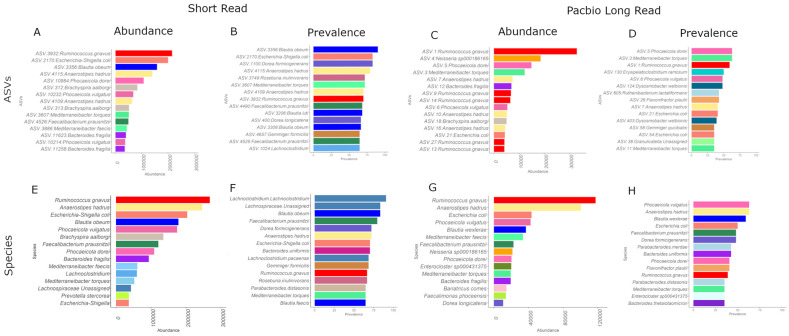
Top 15 ASVs for (**A**) SR abundance, (**B**) SR prevalence, (**C**) PB-LR abundance, (**D**) PB-LR prevalence. Top 15 species for (**E**) SR abundance, (**F**) SR prevalence, (**G**) PB-LR abundance, and (**H**) PB-LR prevalence.

The SR and PB-LR data sets were compared by examining the Top 15 identifiers at the ASV level and agglomerating to species level (Figure 3). At the species level, both SR and PB-LR data sets showed high abundances of *Ruminococcus gnavus*, *Anaerostipes hadrus*, *Escherichia coli*, *Blautia obeum* and *Blautia wexlerae*, *Phocaeicola vulgatus*, *Phocaeicola dorei*, *Mediterraneibacter faecis*, *Mediterraneibacter torques*, *Bacteroides fragilis* and *Faecaliacterium prausnitzii*. The SR data alone included *Brachyspira aalborgi*, *Prevotella stercorea*, *Lachnoclostridium* and *Gemmiger formicilis*. The PB-LR data alone included *Neisseria.sp000186165*, *Enterocloster sp000431375*, *Bariatricus comes*, *Faecalimonas phoceensis* and *Dorea longicatena*. Therefore, there was reasonable concordance between data sets using species appearance in the top 15 ASV abundances. 

In a similar manner, both the SR and the PB-LR data showed the highest prevalence (detection = 0.15) (Figure 3) for *Blautia obeaum* and *Blautia wexlerae*, *Anaerostipes hadrus*, *Faecalibacterium prausnitzii*, *Dorea formicigenerans*, *Ruminococcus gnavus*, *Escherichia coli*, *Mediterraneibacter torques*, *Parabacteroides distasonis* and *Parabacteroides merdae*, *Bacteroides ovatus* and *Bacteroides thetaiotaomicron* and *Parabacteroides distasonis*. The SR data alone were prevalent for *Lachnoclostridium*, *Gemmiger formicilis*, and *Roseburia inulinivorans*. The PB-LR alone were prevalent in *Phocaeicola vulgatus*, *Flavonifractor plautii*, and *Enterocloster sp000431375*. Overall, the species-level agglomerated data showed a Kendall’s W correlation rank concordance of 0.73.

### 3.4. Alpha Diversity Analyses

**Figure 4:**
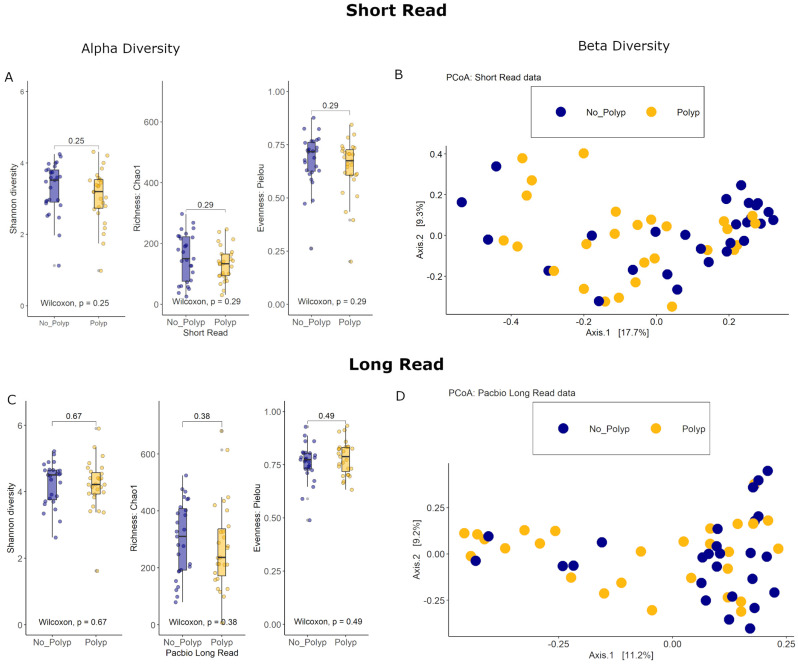
Short-read data: (**A**) Alpha diversity: Box plots of the Shannon diversity index, Chao1 richness estimator, and Pielou evenness index (**B**) Beta diversity: PCoA of the Bray–Curtis distance measure. PacBio long-read data: (**C**) Alpha diversity: Box plots of the Shannon diversity index, Chao1 richness estimator, and Pielou evenness index. (**D**) Beta diversity: PCoA of the Bray–Curtis distance measure. All data colored by polyp status.

The bacterial species diversity for polyp and polyp-free mucosal specimens was assessed for both SR and PB-LR data. There were no statistically significant differences in the Shannon diversity index between these groups. Similarly, there were no statistically significant differences in the Chao1 richness estimator or Pielou’s evenness index (Figure 4). These results suggest that both the number of bacterial species and the distribution of species abundances within cases were similar between the polyp and polyp-free groups for both SR and PB-LR sequencing data.

### 3.5. Beta Diversity Analyses

Differences in the overall taxonomic composition between polyp and polyp-free patient samples were visualized with a Principal Coordinates Analysis using Bray–Curtis distance measures for both SR and PB-LR data (Figure 4). A permutational multivariate analysis of variance (PERMANOVA) showed an R^2^ of 2.4% (*p* = 0.21) for the SR data and an R^2^ of 9.29% (*p* = 0.032) for the PB-LR data.

### 3.6. PERMANOVA

We examined whether covariates known to influence microbiome composition had an impact on bacterial community variation between polyp status. A PERMANOVA performed on the SR data indicated that gender (R^2^ = 5.1%, *p* = 0.002), age (R^2^ = 2.81%, *p* = 0.083), BMI (R^2^ = 2.89%, *p* = 0.080), and polyp status (R^2^ = 1.7%, *p* = 0.45) did not contribute statistically significant explanatory power to the variance in the data. Similarly for the PB-LR data, when considering gender (R^2^ = 2.03%, *p* = 0.896), age (R^2^ = 5.97%, *p* = 0.117), and BMI (R^2^ = 2.04%, *p* = 0.422), the explanatory power of polyp status on bacterial variance was no longer statistically significant (R^2^ = 6.7%, *p* = 0.096). 

### 3.7. Community Composition

**Figure 5:**
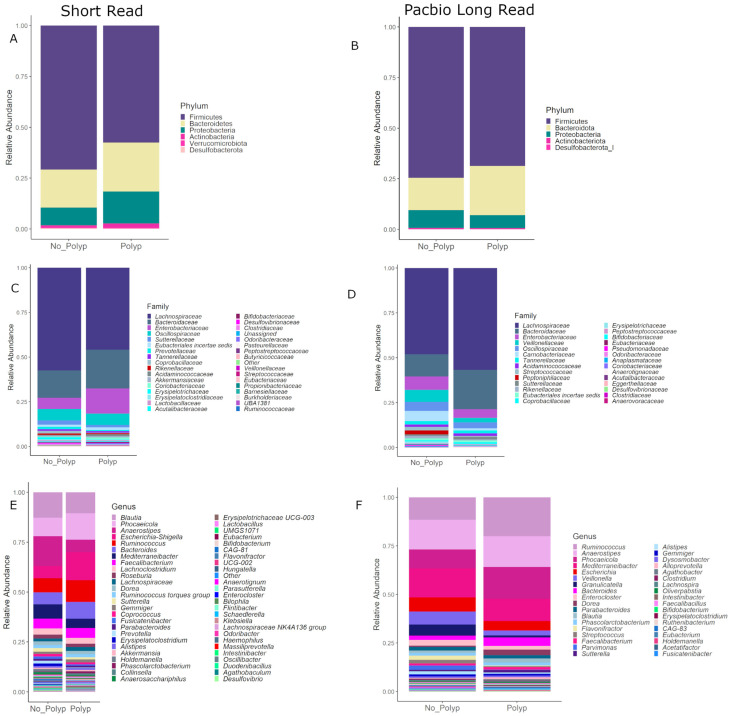
Composition plots with ASVs aggregated at the phylum level for (**A**) SR and (**B**) PB-LR, set at the family level for (**C**) SR and (**D**) PB-LR data, and set at the genus level for (**E**) SR and (**F**) LR data, comparing polyp-associated and polyp-free cases.

Figure 5 shows the taxonomic compositions at the phylum, family and genus levels of taxonomy for both data sets with a detection level set to 0.005. Both data sets suggest that bacteria from the phylum Firmicutes are relatively more abundant in polyp-free mucosal samples (75% SR, 80% LR), whereas the Bacteroidetes/Firmicutes ratio decreased in polyp mucosal samples. At the family level of taxonomy, the *Lachnospiraceae* differ between the SR and PB-LR data; however, both sequencing methods suggest a relative increase in *Bacteroidaceae* in the polyp-associated samples. At the genus level, both data sets suggest a relative increase in *Phoceicola*, *Ruminococcus* and *Bacteroides* in the polyp samples as well as a relative increase in *Mediterraneibacter* in the polyp-free samples.

### 3.8. Phylogenetic Trees

**Figure 6:**
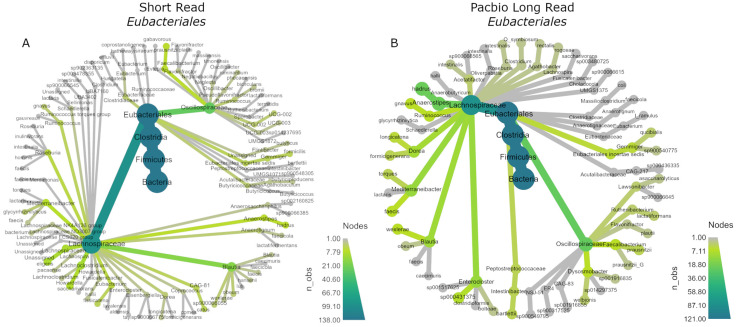
Phylogenetic trees of the order *Eubacteriales* for (**A**) SR and (**B**) PB-LR data.

The data were then subset to the order *Eubacteriales*, as this order was dominant in both sequencing methods and showed the largest differences. The *R* package *metacoder* (Version 0.3.6) was used to display their phylogenetic trees (Figure 6). Both were abundant in *Oscillospiraceae* and *Lachnospiraceae*; however, within the *Lachnospiracaea* family, the SR data assigned more ASVs to *Blautia* and assigned ASVs to more genera overall compared to PB data, while the PB taxonomy assignment was arguably more conservative and assigned more ASVs to *Anaerostipes* compared to *Blautia* and less genera overall. It is also notable that the SR data assigned ASVs to *Clostridiaceae*, whereas the PB data assigned relatively few.

### 3.9. Differential Abundance Analyses

**Figure 7:**
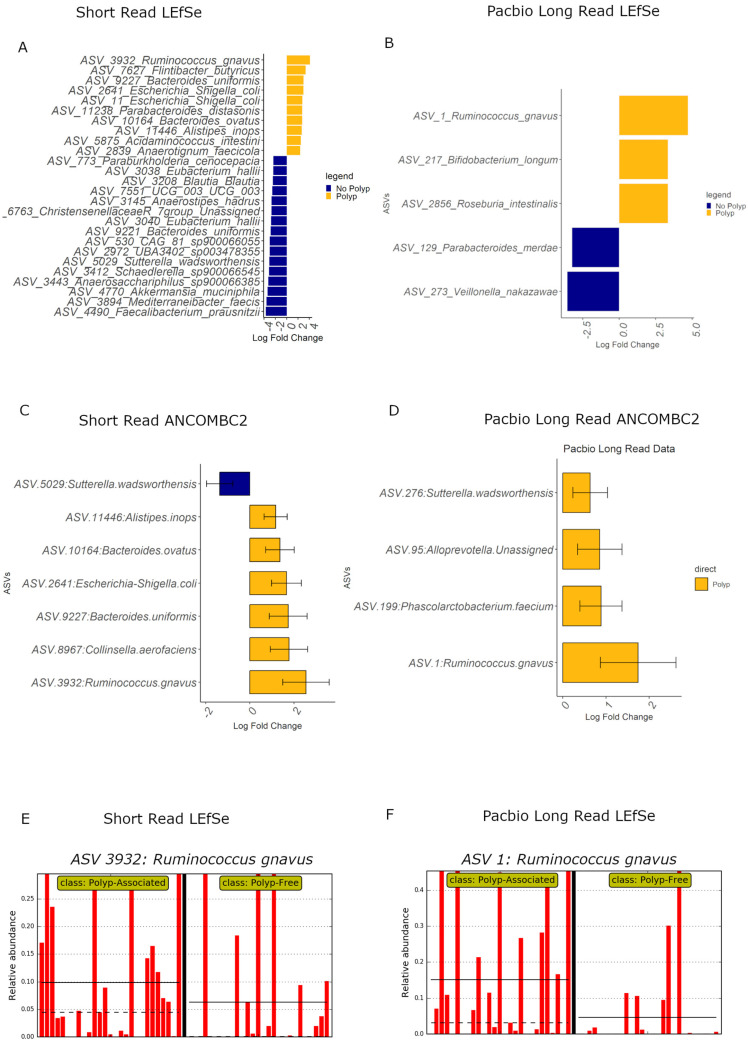
LEfSe differential abundance plot for (**A**) SR and (**B**) PB data sets, and ANCOMBC2 analyses for (**C**) SR and (**D**) PB-LR data, showing bacteria differentially abundant (*p* < 0.05) between polyp-associated and polyp-free samples. LEfSe bar plots for *Ruminococcus gnavus* are displayed for (**E**) SR and (**F**) PB-LR data. Each red bar indicates the relative abundance for a participant specimen. The black vertical bar separates the polyp specimens from polyp-free specimens. The black solid and dashed horizontal bars indicate the mean and median relative abundance for the two patient groups, respectively.

Given that the community composition plots suggest some bacteria show differences in relative abundance between polyp and polyp-free patient specimens, both data sets underwent LEfSe and ANCOMBC2 differential abundance analyses to test for associations of ASVs between the two groups (Figure 7). There was little concordance in the identified ASVs between the SR and PB-LR data sets; however, both sequencing methods identified *Ruminococcus gnavus* as relatively more abundant in polyp patients in both the LEfSe and ANCOMBC2 analyses. Bar plots from the LEfSe analysis show that *Ruminococcus gnavus* is a highly prevalent bacteria, indicating that this result is not simply due to a one- or two-sample abundance bias. 

### 3.10. Multivariate Analyses

The microbiome is an interactive community that communicates through signaling molecules and biofilm formation and may interact in both healthy and disease states. Therefore, relevant ASVs in the early polyp environment may be clarified by employing multivariate analyses, which can identify ASVs that show similar patterns of variance across the data.

**Figure 8:**
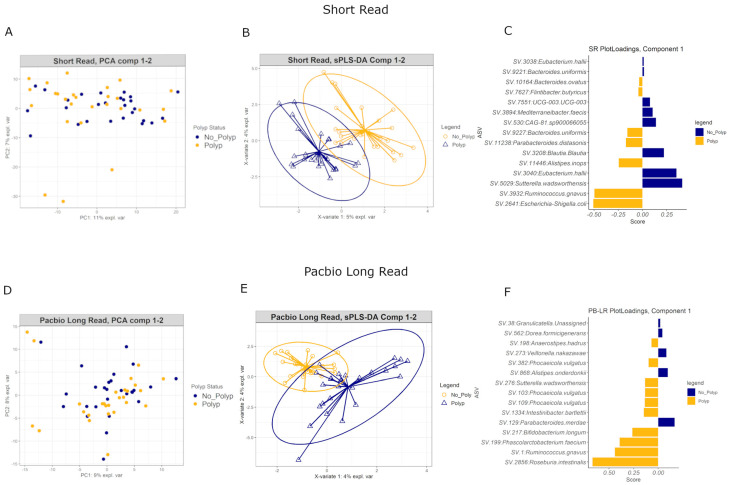
Principal Components Analysis for (**A**) short-read and (**D**) PacBio long-read data. Sparse Partial Least Squares Discriminant Analysis for (**B**) short-read and (**E**) PacBio long-read data. Each plot is colored by polyp status. Bar plots for (**C**) short-read and (**F**) PacBio long-read data: ASVs are ranked according to how influential they are in contributing to the variability in component 1 with the most important on the bottom. The color of each bar corresponds to which group (polyp or polyp-free) has the higher median for the ASV.

Principal Components Analysis of both SR and PB-LR data showed no overall differences in the variance between polyp and polyp-free groups (Figure 8A,D). A supervised Partial Least Squares Discriminant Analysis (sPLS-DA) identified several ASVs that were able to discriminate polyp-associated from polyp-free mucosa samples (Figure 8B,E). Figure 8C,F show the top 15 ASVs for SR and PB-LR data, respectively, that are most influential in contributing the variability in component 1. The performance of these classifications averaged 40–55% error rates, suggesting that these classification algorithms may not generalize to other data sets. In general, the sPLS-DA classification algorithm produced slightly lower error rates on the SR data set compared to the PB-LR data set. However, these multivariate results showed consistency with the univariate differential abundance results. For example, the PB-LR data were consistent in showing *Ruminococcus gnavus*, *Roseburia intestinalis*, *Bifidobacterium longum* and *Sutterella wadsworthensis* as elevated in polyp cases and *Parabacteroides merdae* and *Veillonella nakazawae* elevated in polyp-free cases. The SR data were consistent in showing *Ruminococcus gnavus*, *Escherichia-Shigella*, *Parabacteroides distasonis*, *Bacteroides uniformis* and *ovatus*, and *Alistipes inops* as elevated in polyp cases while *Sutterella wadsworthensis*, *Eubacterium hallii*, *Blautia* and *Mediterraneibacter faecis* were more abundant in polyp-free cases. The SR and PB-LR both identified *Ruminococcus gnavus* as more abundant in polyp cases in both SR and PB-LR data sets in this multivariate analysis.

## 4. Discussion

Previous literature has established a link between stool microbiome composition and colorectal cancer [31,32,33,34,35]; however, less is known about the microbial composition directly at the gut mucosa in early adenoma development. This study examined microbial communities from gut mucosal biopsies adjacent to low-grade bowel polyps by employing 16S rRNA short-read sequencing and PacBio long-read sequencing on the same DNA samples.

Overall, both polyp and polyp-free samples showed similar microbial community diversity (Figure 4), and there were no large differences in bacterial diversity between patients with or without early-stage bowel polyps based on both SR and PB-LR data sets (Figure 4). Composition plots (Figure 5) derived from both SR and PB-LR data sets suggest ASVs from the family *Bacteroidaceae* were enriched in polyp-associated cases, and this was reflected at the genus level, which suggested a greater relative abundance of *Phocaeicola* in the polyp cases. *Rumminococcus* from the family *Oscillospiracaea* was also more abundant in mucosa with polyps compared to patients without polyps. In the SR data, both differential abundance (Figure 7) and multivariate analyses (Figure 8) identified *Ruminococcus gnavus*, *Escherichia-Shigella*, *Bacteroides uniformis*, *Parabacteroides distasonis* and *Alistipes inops* as relatively more abundant in the mucosa of patients with polyps, while the PB data identified *Ruminococcus gnavus*, *Bifidobacterium longum* and *Roseburia intestinalis*. 

The relative increase in abundance of *Ruminococcus gnavus* in polyp patients agrees with studies that found a positive association between *R. gnavus* and *KRAS* mutations in aberrant crypt foci [36], which are very early dysplastic clusters that can form neoplasms. *R. gnavus* has also been found overgrown with *Escherichia Shigella* in biofilms in the proximal mucosa of patients with irritable bowel syndrome (IBS) and ulcerative colitis (UC) [37]. These two bacteria, together with *Collinsella*, which has also been found to be more abundant in mucosal biopsies of adenoma patients and more advanced CRC patients [38], have been associated with inflammation in IBD [39]. *Bacteroides ovatus* has been reported to increase in stool samples of individuals with colorectal adenomas, colorectal cancers and ulcerative colitis [40]. Other species of *Alistipes*, such as *A. finegoldii*, have been shown to contribute to inflammation and colorectal tumor formation in mice [41], and our data highlight a potential role for *A. inops* in early bowel neoplasia. Overall, these results suggest that the bowel mucosal environment in patients with polyps is an inflammatory niche. 

The SR data set also showed a relatively greater abundance of ASVs associated with several butyrate-producing bacteria in polyp-free mucosa samples, including *Faecalibacterium prausnitzii*, *Mediterraneibacter faecis*, *Blautia*, and *Eubacterium hallii*, which is consistent with previous literature that identified butyrate as a protective metabolite in the gut [42,43,44,45]. Our finding of an increased abundance of *Christsensenellaceae R-7 group* in polyp-free mucosa samples agrees with other studies [46,47,48], as does the finding of increased *Veillonella nakazawae*, which is a microbe common to oral cavity biofilms [49]. While members of the *Veillonella* genus have been identified as enriched in stool samples of polyp-free patients, and members of *Veillonella nakazawae* have been identified as enriched in stool samples of patients with metastatic CRC [50,51], to our knowledge, *Veillonella nakazawae* has not previously been known as a component of the microbiome associated with a healthy gut. 

LEfSe and multivariate analysis of the PB-LR data suggested *Bifidobacterium longum*, which is thought to suppress cancer development [52,53], and *Roseburia intestinalis*, a noted butyrate producer, were more abundant in polyp samples, which could not be confirmed with the SR data. Meanwhile, *Parabacteroides merdae*, which was detected in polyp-free samples, has previously been found to be elevated in stool samples of adenoma patients [46,54], although it is decreased in other cancers, such as breast cancer [55]. Similarly, *Alistipes onderdonkii*, elevated in polyp-free patients in our study, was increased in the tumor tissue of colorectal cancer patients compared to adjacent tissue in previous studies [56]. Moreover, ASVs associated with *Sutterella wadsworthensis* were more abundant in polyp-free patient mucosa in the short-read data yet increased in polyp-associated samples in the Pacbio long-read data. We considered that this discrepancy could result from misidentification of the short reads assigned to *S. wadsworthensis*. However, this was unlikely, as the top results for a selection of the four most abundant of these reads in the NCBI nucleotide Basic Local Alignment Search Tool (nBLAST) were indeed *S. wadsworthensis*, with a percent identity ranging from 99.75% to 100% (E value = 0). Previous studies in the literature suggest that *S. wadsworthensis* tends to be more abundant in IBS [57] stool samples of FOBT+ individuals [58] and in stool samples of individuals with pre-cancerous or CRC lesions [59], which are findings consistent with our long-read data obtained from mucosal specimens. Overall, these results suggest that different species or strains may have different effects on polyp development or may differ in effect depending on their interactions with other bacteria in the environment.

These results need to be interpreted in the context of some limitations. Although our PERMANOVA and ANCOMBC differential abundance analyses included covariates known to influence bacterial composition (age, gender and BMI) [60], other variables, including diet and alcohol consumption, smoking, and the use of medications other than antibiotics were not available. Moreover, inherent in 16S rRNA short-read sequencing is the inability to resolve most microbes to the species and strain level of taxonomic classification, as highlighted in Figure 3, where differences in ASV abundances are noted compared to long-read 16S sequencing, but these could be resolved by agglomerating to the species level for analysis. Long-read 16S sequencing improves this issue, although we encountered problems when sequencing the entire 16S-ITS-23S genomic region due to substantial human DNA interference from biopsies. The issue of human DNA enrichment was overcome by targeting the V1–V9 16S rRNA region PCR product, which enabled species-level classification in 90% of reads. Moreover, the concordance between the short-read and long-read data sets in differentially abundant ASVs was limited, which is consistent with other comparisons of these two technologies [20,61,62]. This may be due to the differences in sequencing technologies as well as bioinformatics pipelines employed. Efforts were made to minimize this by employing QIIME2, DADA2 and the same taxonomic databases for both data sets as well as addressing discrepancies in the three taxonomic databases by employing manual curation. This lack of overlap in identified ASVs highlights the challenge of cross-study comparisons.

## 5. Conclusions

To our knowledge, this is the first study to compare short-read and long-read 16S rRNA sequencing of mucosal bacteria from patients with and without bowel polyps. Long-read sequencing increased the number of ASVs identified to the species level to 90% compared to short-read sequencing at 50%; however, our study highlighted a disparity in the results when attempting to compare SR-and LR data. Despite this, both analyses using SR and LR data showed that *Ruminococcus gnavus* was more abundant in polyp cases compared with poly-free controls, even though these patients had not received a diagnosis of IBD, which is where *R. gnavus* is typically noted. Combined with elevated mucosal detection of *E. coli*, *Collinsella*, *B. ovatus*, and *P. feasis* in polyp patients, this suggests that early bowel polyps evolve in a local inflammatory niche. *V. nakazawei*, *F. prausnitzii*, *Blautia*, *E. hallii* and *M. faecis* appear to have a protective effect, and a common feature for most of these microorganisms is their ability to produce butyrate. Further studies are necessary to confirm and elucidate whether these microbes contribute to bowel tumorigenesis.

## Figures and Tables

**Table 1:** Colonoscopy patient demographics.

Characteristic			Polyp Patients	Polyp-Free Patients	*p*-Value
N			27	27	
Age			68 (27–86) ^1^	57 (37,82) ^1^	0.11 ^2^
Gender	Female		9	9	0.8 ^3^
	Male		18	18	
BMI			25 (19,30) ^1^	25 (23,31) ^1^	0.2 ^2^
Type of Lesion	SL	<10mm	1	0	
		≥10mm	0	0	
	TA	<10mm	22	0	
		≥10mm	2	0	
	TVA	<10mm	0	0	
		≥10mm	2	0	
Indications for colonoscopy	Surveillance—1st degree relative Polyp or CRC		2	4	0.7 ^3^
	Surveillance—Personal History Polyp or CRC		11	8	
	Rectal bleeding		7	10	
	FOBT+/FIT+		4	2	
	Abnormal Bowel Symptoms		2	1	
	Other		1	2	

^1^ Range ^2^ Wilcoxon rank-sum test; ^3^ Pearson’s Chi-squared test. SL = serrated lesion, TA = tubular adenoma, TVA = tubulovillous adenoma. FOBT+/FIT+ = fecal occult blood test/fecal immunochemical test positive. Other = iron deficiency anemia (1), Neuroendocrine tumour in appendix (1 participant), Recurrent small bowel obstruction (1 participant).

**Table 2:** Comparison of sequencing counts for SR and PB-LR data sets.

Sequencing Counts	Short Read	PacBio Long Read
Total	20,352,406	4,784,897
Average (median) per sample	96,504 (97,959)	27,367 (27,545)
Average (median) per sample—post-filter, non-chimeric	61,305 (60,287)	19,205 (19,999)
Average (median) per sample—post filter, non-chimeric (%)	64.16 (63.29)	72.25 (78.42)

## Data Availability

Sequencing data and associated meta-information have been deposited in the NCBI Bioprojects archive PRJNA1013750.
